# Modelling the Cost-Effectiveness of Hepatitis A in South Africa

**DOI:** 10.3390/vaccines12020116

**Published:** 2024-01-24

**Authors:** Jenna Patterson, Susan Cleary, Jared Michael Norman, Heiletjé Van Zyl, Timothy Awine, Saadiyah Mayet, Benjamin Kagina, Rudzani Muloiwa, Gregory Hussey, Sheetal Prakash Silal

**Affiliations:** 1 Vaccines for Africa Initiative, School of Public Health, University of Cape Town, Cape Town 7925, South Africa; benjamin.kagina@uct.ac.za (B.K.); gregory.hussey@uct.ac.za (G.H.); 2 Institute of Infectious Disease and Molecular Medicine, University of Cape Town, Cape Town 7925, South Africa; 3 School of Public Health, University of Cape Town, Cape Town 7925, South Africa; susan.cleary@uct.ac.za; 4 Modelling and Simulation Hub, Africa (MASHA), Department of Statistical Sciences, University of Cape Town, Cape Town 7700, South Africa; jared.norman@uct.ac.za (J.M.N.); vzyhei003@myuct.ac.za (H.V.Z.); timothy.awine@navrongo-hrc.org (T.A.); saadiyah.mayet@gtc.ox.ac.uk (S.M.); 5 Department of Paediatrics and Child Health, Red Cross War Memorial Children’s Hospital, University of Cape Town, Cape Town 7700, South Africa; rudzani.muloiwa@uct.ac.za; 6 Centre for Global Health, Nuffield Department of Medicine, Oxford University, Oxford OX3 7LG, UK

**Keywords:** hepatitis A vaccine, mathematical model, cost-effectiveness

## Abstract

The World Health Organization (WHO) recommends the consideration of introducing routine hepatitis A vaccination into national immunization schedules for children ≥ 1 years old in countries with intermediate HAV endemicity. Recent data suggest that South Africa is transitioning from high to intermediate HAV endemicity, thus it is important to consider the impact and cost of potential routine hepatitis A vaccination strategies in the country. An age-structured compartmental model of hepatitis A transmission was calibrated with available data from South Africa, incorporating direct costs of hepatitis A treatment and vaccination. We used the calibrated model to evaluate the impact and costs of several childhood hepatitis A vaccination scenarios from 2023 to 2030. We assessed how each scenario impacted the burden of hepatitis A (symptomatic hepatitis A cases and mortality) as well as calculated the incremental cost per DALY averted as compared to the South African cost-effectiveness threshold. All costs and outcomes were discounted at 5%. For the modelled scenarios, the median estimated cost of the different vaccination strategies ranged from USD 1.71 billion to USD 2.85 billion over the period of 2023 to 2030, with the cost increasing for each successive scenario and approximately 39–52% of costs being due to vaccination. Scenario 1, which represented the administration of one dose of the hepatitis A vaccine in children < 2 years old, requires approximately 5.3 million vaccine doses over 2023–2030 and is projected to avert a total of 136,042 symptomatic cases [IQR: 88,842–221,483] and 31,106 [IQR: 22,975–36,742] deaths due to hepatitis A over the period of 2023 to 2030. The model projects that Scenario 1 would avert 8741 DALYs over the period of 2023 to 2030; however, it is not cost-effective against the South African cost-effectiveness threshold with an ICER per DALY averted of USD 21,006. While Scenario 3 and 4 included the administration of more vaccine doses and averted more symptomatic cases of hepatitis A, these scenarios were absolutely dominated owing to the population being infected before vaccination through the mass campaigns at older ages. The model was highly sensitive to variation of access to liver transplant in South Africa. When increasing the access to liver transplant to 100% for the baseline and Scenario 1, the ICER for Scenario 1 becomes cost-effective against the CET (ICER = USD 2425). Given these findings, we recommend further research is conducted to understand the access to liver transplants in South Africa and better estimate the cost of liver transplant care for hepatitis A patients. The modelling presented in this paper has been used to develop a user-friendly application for vaccine policy makers to further interrogate the model outcomes and consider the costs and benefits of introducing routine hepatitis A vaccination in South Africa.

## 1. Introduction

### Background

Over the last two decades, Southern Africa has been considered to have high hepatitis A virus (HAV) endemicity with seroprevalence ≥ 90% by 10 years old [1]. Data suggest, however, that South Africa has transitioned from high to intermediate or low hepatitis A virus endemicity with fewer children acquiring hepatitis A infection and developing natural immunity at a young age [2]. With this shift and a rise in the age of people susceptible to HAV infection in the population, the risk for serious outbreaks and a significant burden of the disease increases.

The World Health Organization (WHO) recommends the consideration of introducing routine hepatitis A vaccination into national immunization schedules for children ≥ 1 years old in countries with intermediate HAV endemicity. Previously published studies have found routine hepatitis A vaccination strategies to be cost-effective in countries with existing childhood immunization programs; however, an analytical framework to assess the impact and cost of different routine hepatitis A vaccination strategies in South Africa has not yet been developed [2,3,4,5,6,7,8,9,10,11,12]. A new dynamic transmission model was deemed necessary to develop so that South Africa’s hepatitis A force of infection could be robustly estimated, and population-level clinical outcome and cost data collected in previous studies could be properly implemented.

While the Expanded Program on Immunization in South Africa (EPI-SA) has been a leader in adopting new vaccines on the African continent, there are considerable economic obstacles facing the introduction of new vaccines into the EPI-SA. The implementation of new vaccines requires a large upfront investment, and the success of new vaccination programs is often uncertain in low- and middle-income countries (LMICs). In countries with health budgets that have little room for expansion, it is important for economic evaluations to deliver strong evidence for opportunities of cost-effectiveness. We evaluated the cost, outcomes, cost-effectiveness of different potential routine hepatitis A vaccination strategies in South Africa. This model was developed with the aim to support the South African National Advisory Group on Immunization (NAGI) Hepatitis A Working Group’s consideration of introducing routine hepatitis A vaccination into the EPI-SA.

## 2. Methods

### 2.1. Transmission Model

**Figure 1:**
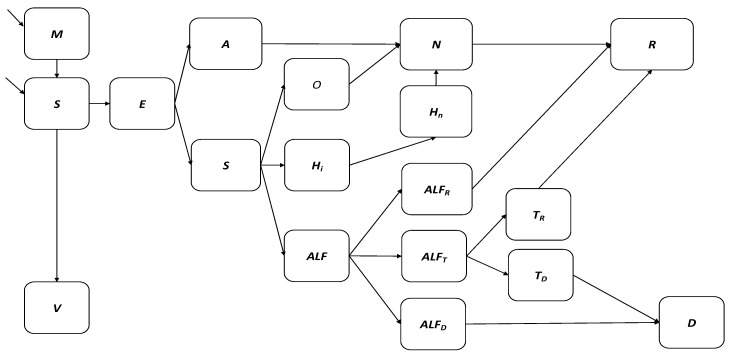
Flow diagram of hepatitis A transmission and vaccination model.

Ordinary differential equations were used to develop an age-structured model for hepatitis A transmission dynamics in South Africa. The model diagram is displayed in Figure 1 and the differential equations are presented in Supplementary Table S1. In the model, the South African population is divided into 18 distinct hepatitis-A specific epidemiological compartments (Table 1), which are further stratified by 19 age groups (annual ages until 9 years old followed by 5-year age groups). The population is modelled over time through the birth rate, aging rate, and age-specific death rate.

Figure 1 depicts the hepatitis A dynamic transmission model where the South African population is divided into 18 distinct hepatitis-A specific epidemiological compartments. Births are classified according to the presence of maternal antibodies into the M and S compartments. The V compartment represents hepatitis A vaccination. Hepatitis A infection occurs in the E compartment. The A (asymptomatic) and Sy (symptomatic) compartments represent active hepatitis A infections. The O and Hi compartments represent the treatment sought for uncomplicated hepatitis A cases, while the ALF compartment represents the treatment sought for viral-induced acute liver failure. Acute liver failure cases spontaneously recover from liver injury into compartment ALFR, indicate the need for liver transplant and move into compartment ALFT, or die due to liver injury without transplant in compartment ALFD. Liver transplant cases recover in compartment TR or die following the transplant procedure in compartment TD. The N compartment represents previous hepatitis A cases with anti-HAV IgG antibodies that may still have present anti-HAV IgM antibodies while R represents fully recovered hepatitis A cases with anti-HAV IgG antibodies and no anti-HAV IgM antibodies. Lastly, D represents all death due to hepatitis A infection.

Births are classified according to the presence of maternal antibodies (*propM*) into the *M* (maternal antibody) and *S* (susceptible) compartments. The *V* compartment represents hepatitis A vaccination where vaccinated individuals develop anti-HAV IgG antibodies at the rate of gamma. Hepatitis A infection occurs in the *E* compartment with the age-specific force of infection given by:

lambda_i_ = β_ij_ × I/P × Prel × betaE_i_ × prevE × Erel;

where infection is determined by the number of contacts, the proportion of infected contacts, the transmission probability per contact, the environmental presence of HAV, and the nature of mixing between age groups. The contact pattern between age groups is determined by the conditional probability contact matrix β_ij_ for South Africa adapted from Prem et al. 2017 (Supplementary Table S2) [13].

The *A* (asymptomatic) and *Sy* (symptomatic) compartments represent active hepatitis A infections with anti-HAV IgM antibodies following an incubation period *nu*. *O* and *H_i_* represent the treatment sought for uncomplicated hepatitis A cases, while the *ALF* compartment represents the treatment sought for viral-induced acute liver failure. Acute liver failure cases spontaneously recover from liver injury into compartment *ALF_R_*, indicate the need for liver transplant and move into compartment *ALF_T_*, or die due to liver injury without transplant in compartment *ALF_D_*. Liver transplant cases recover in compartment *T_R_* at rate *gammaT* or die following the transplant procedure in compartment *T_D_* at rate *TDrate*. Hospitalized and outpatient cases lose infectivity at the rate of *gamma* and move into the *N* compartment representing previous hepatitis A cases with anti-HAV IgG antibodies that may still have present anti-HAV IgM antibodies. *R* represents fully recovered hepatitis A cases with anti-HAV IgG antibodies and no anti-HAV IgM antibodies, while *D* represents all death due to hepatitis A infection.

### 2.2. Model Calibration

The model is fitted to the annual South African hepatitis A seroprevalence (anti-HAV IgG) data between 2005 to 2015 from the National Institute of Communicable Diseases (NICD) [2,14]. Ethical approval for the use of this data was obtained from the National Institute of Communicable Diseases (NICD) and Institutional Review Board approval was obtained from the University of Cape Town Human Research Ethics Committee (FSREC 106—2019). The observed rising trend in hepatitis A seroprevalence data suggests an increase in the incidence of hepatitis A infections (anti-HAV IgM) in South Africa across all age groups. The increase in hepatitis A seroprevalence, however, is not enough to reach the definition of high HAV endemicity as seroprevalence remains <90% for children and adolescents < 15 years old between 2005–2015.

**Figure 2:**
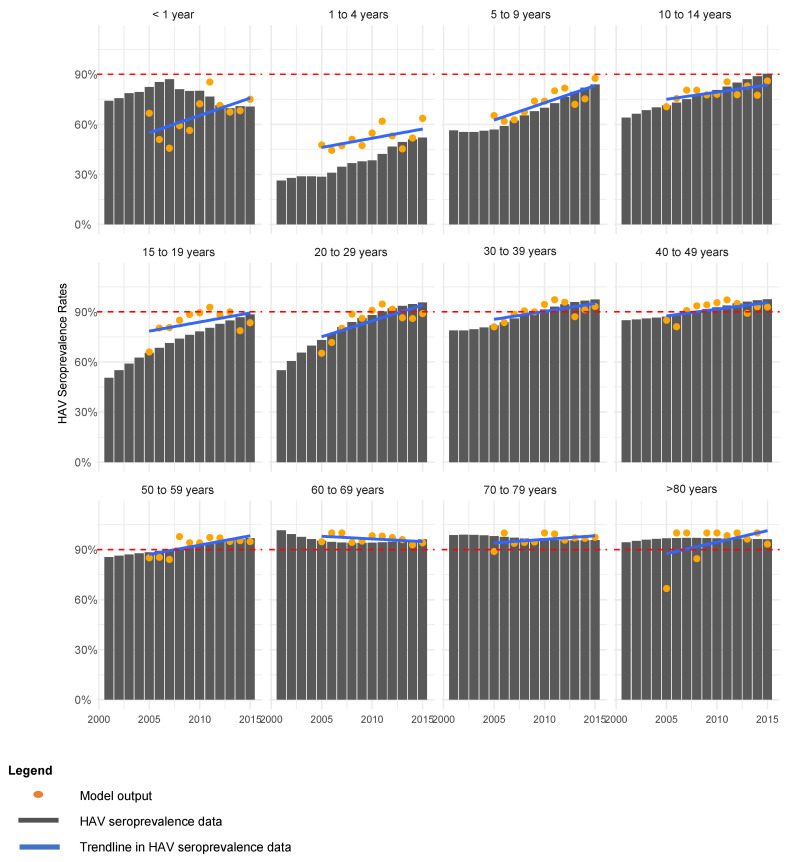
Model fitting to HAV seroprevalence (anti-HAV IgG) data by age group.

The model was run from 2000 with parameters in Table 2 to reach a steady state before being fitted through maximum likelihood estimation to the seroprevalence data from 2005 to 2015. The incidence of HAV seroprevalence in 2015 was considered the baseline for future predictions and all parameters from 2015 were held constant for scenario testing. The NICD seroprevalence data and model seroprevalence outputs are compared by age group in Figure 2.

Owing to uncertainty in the dataset and a large number of unknown parameters, a simulation approach was selected for data fitting. We simulated 100,000 Latin hypercube sampled parameter combinations to calibrate the model to key features in the dataset. As the South African testing volumes, IgM positivity rates, and age specific anti-HAV seroprevalence rates varied by year, the model was calibrated to three conditions (features) estimated from the NICD seroprevalence data. As the volume of anti-HAV total antibody tests and proportion of positive total antibody results was highest in 2011, this was chosen as the most reliable year of reporting [14]. Only those parameter sets from model runs that reproduced the following criteria were deemed suitable for further analysis:

- Seroprevalence below 90% for individuals < 20 years old between 2005–2015; and
- Seroprevalence to only reach ≥90% in individuals 20–29 years old in 2011 and 2012; and
- Seroprevalence below 60% for individuals < 5 years old after 2012.

**Figure 3:**
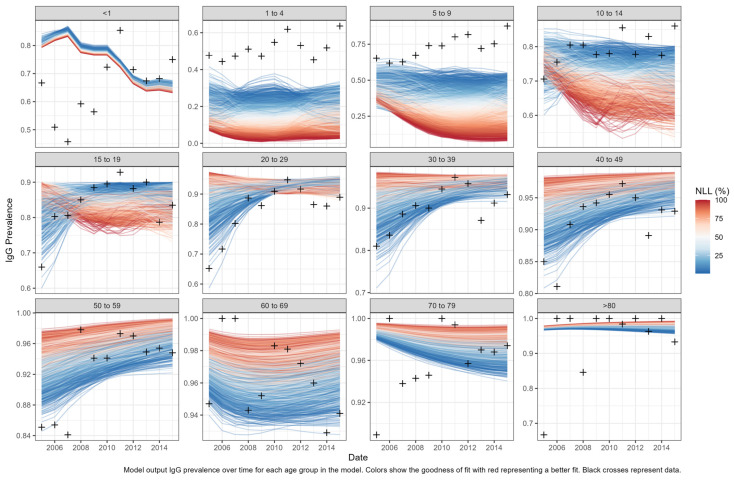
Model calibration negative log likelihood results. Model output IgG prevalence over time for each age group in the model. Colors show the goodness of fit with red representing a better fit. Black crosses represent data.

We accepted 1513 of the 100,000 parameter combinations used to simulate the model reproduced the epidemiological criteria above. The calibration negative log likelihood results are displayed in Figure 3.

### 2.3. Scenario Analyses

We used the calibrated model with accepted parameter sets to evaluate various hepatitis A vaccination scenarios from 2023 to 2030. Nested vaccination scenarios were built in line with existing hepatitis A immunization strategies in LMICs and feedback South African National Advisory Group on Immunization’s (NAGI’s) Hepatitis A Working Group. We assessed how each scenario impacted the number of symptomatic hepatitis A cases, hepatitis A mortality, total costs, and total DALYs as compared to the baseline of no vaccination until 2030. The median values are reported for all model outcomes with associated interquartile ranges. In each scenario, the administration of vaccine doses 1 and 2 began in 2023 and catch-up doses began in 2027. The vaccination coverage rates were assumed to be equal to average performance estimates of the EPI-SA in 2019 in relevant age groups and were estimated to be 80%, 60%, and 40% for dose 1, dose 2, and catch-up doses, respectively [26]. Vaccine efficacy estimates taken from published literature for dose 1 and subsequent doses were estimated to be 98% and 95%, respectively [27]. As the vaccination scenarios are designed based on age groups, vaccination is designed to occur instantaneously as a child ages into a relevant group.

Baseline Scenario: No vaccination.

Scenario 1: Dose 1 administered in children < 2 years old.

Scenario 2: Dose 1 administered in children < 2 years old + Dose 2 administered in children < 3 years old.

Scenario 3: Dose 1 administered in children < 2 years old + Dose 2 administered in children < 3 years old + Catch-up dose administered in children < 5 years old.

Scenario 4: Dose 1 administered in children < 2 years old + Dose 2 administered in children < 3 years old + Catch-up dose administered in children < 10 years old.

### 2.4. Estimation of Hepatitis A Treatment and Routine Immunization Costs

We conducted the economic evaluation in accordance with CHEERS guidelines [28]. We adopted a provider’s perspective that requires the inclusion of direct health care costs to estimate the cost-effectiveness of the scenarios. The direct costs included treatment costs of HAV and the costs of vaccination. Treatment costs included costs for outpatient care, hospitalization, and liver transplants. Cost inputs displayed in Table 3 were taken from published literature. Where costs were reported in South African Rands (ZAR), they were adjusted to ZAR 2020 using the South African medical consumer price index (CPI) and converted to 2020 United States Dollars (USD) using an average exchange rate over 2020 (USD 1 = ZAR 16.61) [29,30]. Where costs were reported in USD, they were converted to ZAR using the relevant exchange rate and adjusted to ZAR 2020 using the South African medical CPI, and then converted back to USD using the 2020 exchange rate.

The cost inputs displayed in Table 3 for hepatitis A outpatient and inpatient treatment at tertiary healthcare facilities were taken from Patterson et al. 2022 [21]. The cost of liver transplant was broken down into treatment of transplant cases and cost of transplant procedures at tertiary healthcare facilities. The cost of treatment for liver transplant cases was calculated by multiplying the cost per inpatient day equivalent (PDE) (USD 539.86 for patients < 15 years and USD 821.12 for patients ≥ 15 years old) by the average length of stay (LOS) (26 days) [21,31]. The cost of liver transplant was taken from the Department of Health Uniform Patient Fee Schedule (UFPS) 2020 to include the procedure and specialist practitioner fee for liver transplants at public tertiary facilities [32]. We applied an access parameter of 30% to the cost of liver transplant as not all patients who indicate the need for liver transplant in South Africa will receive one due to social contraindications including limited access to healthcare and poverty. To qualify for a transplant, social and socioeconomic criteria are used as exclusion criteria for patients as transplant requires adherence to lifelong treatment and the presence of social support structures for positive outcomes.

Vaccination cost inputs were comprised of the cost per vaccine dose and cost of vaccine administration (clinic visit). The mean cost per vaccine dose was calculated as the average of the single exit prices reported for Havrix junior single dose vial 0.5 mL and Avaxim prefilled syringe 80 0.5 mL [33]. As the vaccination scenarios modelled did not include the administration combined with vaccines in the EPI, the cost per vaccine clinic visit was sourced from the District Health Barometer 2020 Public Health Clinic (PHC) expenditure and added to the cost per dose [34].

The DALY inputs are displayed in Table 4. We calculated disability-adjusted life years (DALYs) by adding the years lived with disability (YLD) and years of life lost (YLL). The YLD were calculated by applying the disease state incidence, disability weight, and time lived in each disease state. The YLL was calculated by applying the number of deaths due to hepatitis A by the remaining life expectancy at time of death. We assumed a disability weight of 0.051 (95% CI 0.032, 0.074) for all outpatient hepatitis A cases based on the Global Burden of Disease Study 2017 disability weigh estimate for moderate acute hepatitis A [35]. We assumed a disability weight of 0.133 (95% CI 0.008, 0.190) for all hospitalized patients based on the Global Burden of Disease Study 2017 disability weigh estimate for severe acute hepatitis A [35]. We assumed a disability weight of 0.54 from all patients with liver transplant based on the Global Burden of Disease Study 2017 disability weight estimate for terminal phase of liver cancer due to hepatitis B infection [35]. Future costs and outcomes (i.e., DALYs) modelled were discounted at 5% as recommended by the Health Technology Assessment (HTA) guidelines in South Africa [36].

The results of the economic evaluation for each scenario are reported as incremental cost-effectiveness ratios (ICERs) calculated by comparing each scenario to the baseline given that the vaccination scenarios were nested scenarios. The cost-effectiveness of scenarios was judged against the South African cost-effectiveness threshold (CET) of USD 3276 per DALY averted [37]. The South African CET reported was reported in 2015 and adjusted to ZAR 2020 using the South African medical CPI and then converted to USD using the 2020 exchange rate.

**Table 3:** Cost inputs.

Cost	Cost (USD 2020)	Source
Outpatient treatment of hepatitis A cases in patients < 15 years	USD 177.88	Patterson et al., 2022 [21]
Outpatient treatment of hepatitis A cases in patients ≥ 15 years old	USD 264.94	Patterson et al., 2022 [21]
Inpatient treatment of hepatitis A cases in patients < 15 years	USD 1856.79	Patterson et al., 2022 [21]
Inpatient treatment of hepatitis A cases in patients ≥ 15 years old	USD 6382.37	Patterson et al., 2022 [21]
Inpatient treatment of liver transplant patients < 15 years	USD 11,337.14	Calculated value based on PDE and LOS
Inpatient treatment of liver transplant patients ≥ 15 years old	USD 21,329.20	Calculated value based on PDE and LOS
Liver transplant procedure (all ages)	USD 1787.74	UPFS 2020 [32]
Dose of paediatric hepatitis A vaccine	USD 19.71	MedicinePrices.org [33]
Clinic visit for vaccine administration	USD 136.15	Massyn et al., 2020 [34]

**Table 4:** DALY inputs.

Variable	Value	Source
DW outpatient hepatitis A cases	0.051	GBD 2018 [35]
DW hospitalized hepatitis A cases	0.133	GBD 2018 [35]
DW liver transplant	0.54	GBD 2018 [35]
YLD hepatitis A outcomes, excluding liver failure (days)	21	Johns Hopkins 2021 [23]
YLD liver transplant (days)	180	Johns Hopkins 2021 [23]

Abbreviations: DW = disability weight; YLD = years lived with disability.

### 2.5. Sensitivity Analyses

We ran several one-way sensitivity analyses on key cost and DALY parameters for the most desirable vaccination scenario. We conducted sensitivity analyses on the baseline scenario to determine how the total costs of the scenario would vary for the below changes in cost assumptions and discount rates and display the results in a tornado diagram.

- Remove the costs of clinic visits for vaccine administration (USD 136.15);
- Vary the access to liver transplant procedures to 0% and 100%;
- Vary the discount rate between 0% and 10%.

## 3. Results

### 3.1. Baseline Scenario

Without the implementation of any hepatitis A vaccination strategy from 2023, hepatitis A seroprevalence (anti-HAV IgG) in children < 10 years old is estimated to reach 95.87% [IQR: 93.42–96.11%] by 2030. However, even with this increase in HAV seroprevalence among children < 10 years old, our model projects that the annual number of symptomatic hepatitis A cases is expected to decline by less than 2% from an expected 49,778 [IQR: 31,546, 87,872] symptomatic cases in 2023 to 48,878 [31,057, 87,067] symptomatic cases in 2030. In addition, our model projects that annual hepatitis A mortality will decline by less than 4% from an expected 11,924 [IQR: 8621–16,446] deaths due to hepatitis A in 2023 to 11,536 [IQR: 8342, 16,076] deaths in 2030.

Table 5 shows the impact of each vaccination scenario on symptomatic hepatitis A cases and mortality over the period of 2023–2030.

Scenario 1: The administration of one dose of the hepatitis A vaccine in children < 2 years old requires approximately 5.3 million vaccine doses over 2023–2030. The model projects Scenario 1 would avert a total of 136,042 symptomatic cases [IQR: 88,842–221,483] and 31,106 [IQR: 22,975–36,742] deaths due to hepatitis A over the period of 2023 to 2030. Under Scenario 1, one symptomatic case would be averted for approximately every 39 vaccines administered. Similarly, one death due to hepatitis A would be averted for approximately every 171 vaccines administered.

Scenario 2: The administration of a first dose of the hepatitis A vaccine in children < 2 years old and a second dose in children < 3 years old requires approximately 7.8 million vaccine doses over 2023–2030. The model projects Scenario 2 would avert a total of 255,857 [IQR: 159,721–225,065] symptomatic cases and 31,585 [IQR: 23,388–37,240] deaths due to hepatitis A over the period of 2023 to 2030. Under Scenario 2, one symptomatic case would be averted for approximately every 56 vaccines administered. Similarly, one death due to hepatitis A would be averted for approximately every 247 vaccines administered.

Scenario 3: The administration of a first dose of the hepatitis A vaccine in children < 2 years old and a second dose in children < 3 years old with a catch-up dose administered to children < 5 years old that are not already vaccinated requires approximately 9.2 million vaccine doses over 2023–2030. The model projects that Scenario 3 would avert a total of 259,318 [IQR: 162,828–477,574] symptomatic cases and 30,982 [IQR: 22,502–37,488] deaths due to hepatitis A over the period of 2023 to 2030. Under Scenario 3, one symptomatic case would be averted for approximately every 68 vaccines administered. Similarly, one death due to hepatitis A would be averted for approximately every 298 vaccines administered.

Scenario 4: The administration of a first dose of the hepatitis A vaccine in children < 2 years old and a second dose in children < 3 years old with a catch-up dose administered to children < 10 years old not already vaccinated requires approximately 11.7 million vaccine doses over 2023–2030. The model projects that Scenario 4 would avert a total of 267,947 [IQR: 169,625–482,796] symptomatic cases and 29,890 [IQR: 21,235–37,309] deaths due to hepatitis A over the period of 2023 to 2030. Under Scenario 4, one symptomatic case would be averted for approximately every 86 vaccines administered. Similarly, one death due to hepatitis A would be averted for approximately every 392 vaccines administered.

### 3.2. Cost-Effectiveness of Vaccination

For the modelled scenarios, the median estimated cost of the different vaccination strategies ranged from USD 1.71 billion to USD 2.85 billion over the period of 2023 to 2030, with the cost increasing for each successive scenario and approximately 39–52% of the costs being due to vaccination. The ICERs for the vaccination scenarios in Table 6 were calculated by comparing each scenario to the baseline. In Supplementary Table S3, we also present ICERS calculated by comparing each scenario to the previous undominated and less costly scenario. The cost-effectiveness of scenarios was judged against the South African CET of USD 3276 per DALY averted [37].

The model suggests that the implementation of all potential vaccination scenarios would deliver health gains in the population, with the lowest incremental cost per DALY averted against baseline for Scenario 1. The model projects that Scenario 1, representing the administration of a single dose of hepatitis A vaccine in children < 2 years old from 2023 to 2030, would avert 8741 DALYs; however, it is not cost-effective against the CET with an ICER per DALY averted of USD 21,006. In Supplementary Table S3, the results of our model show that Scenarios 3 and 4 were absolutely dominated in that they produced less health gains and were more expensive than Scenarios 1 and 2. These results signal that the timing of vaccination is critical in the roll-out of potential hepatitis A prevention programs. While Scenario 3 and 4 include the administration of more vaccine doses and avert more symptomatic cases of hepatitis A, the total health gains are smaller than in Scenarios 1 and 2 owing to the population being infected before vaccination through the mass campaigns at older ages. With our results, the model suggests that natural exposure to HAV may begin as early as 3 years old in South Africa.

### 3.3. Sensitivity Analysis

**Figure 4:**
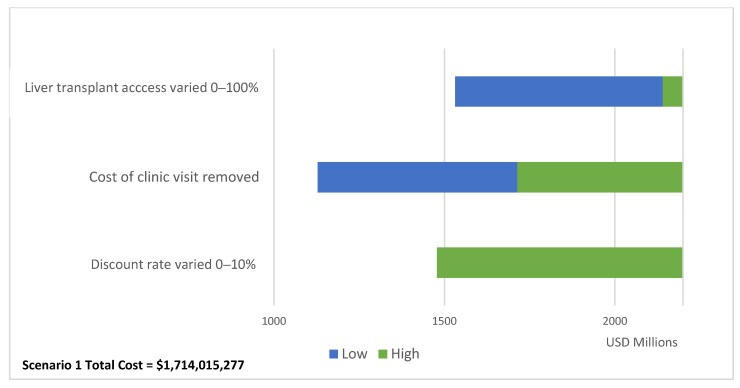
One-way sensitivity analysis impact on Scenario 1 total costs.

Our one-way sensitivity analysis on the total cost of Scenario 1 reported in Figure 4 shows that varying access to liver transplant between 0% and 100% has the largest impact in results (total cost delta = USD 609,302,599). When increasing the access to liver transplant to 100% for baseline and Scenario 1, the ICER for Scenario 1 becomes cost-effective against the CET (ICER = USD 2425) (Supplementary Table S4).

## 4. Discussion

Our results indicate that the administration of a single dose of the hepatitis A vaccine in children < 2 years old in South Africa between the period of 2023 to 2030 would produce significant health gains. The implementation of this vaccination strategy between 2023 and 2030 has the potential to avert a total of 136,042 symptomatic cases [IQR: 88,842–221,483] and 31,106 [IQR: 22,975–36,742] deaths due to hepatitis A. The model projects that for every 39 hepatitis A vaccines administered, one symptomatic case of hepatitis A would be averted. Similarly, for every 171 hepatitis A vaccines administered, one death due to hepatitis A would be averted. Our results show that the implementation of a single dose of the hepatitis A vaccine in children < 2 years old in South Africa would avert 8741 DALYs over the period of 2023–2030, However, is not cost-effective against the South African CET of USD 3276 per DALY averted with an ICER per DALY averted of USD 21,006.

The total cost of implementing a single dose of the hepatitis A vaccine for children < 2 years old over the eight-year intervention period is estimated to be USD 1.71 billion, with approximately 39% of the cost due to the 5.3 million vaccine doses required. When reviewing the total cost of modelled scenarios, it is notable that less than 50% of the total costs were due to vaccination. These results indicate that the burden of hepatitis A in the baseline scenario is heavy for the healthcare system and national health budget in South Africa.

Our study signals that the timing of hepatitis A vaccine administration is important as Scenarios 3 and 4 were absolutely dominated by Scenarios 1 and 2. While Scenario 3 and 4 include the administration of more vaccine doses and avert more symptomatic cases of hepatitis A, the total health gains are less than in Scenarios 1 and 2 owing to the population being infected before vaccination through the mass campaigns at older ages.

In regard to patient outcomes, we applied a liver transplant access parameter of 30% in our economic evaluation as not all patients who indicate the need for liver transplant in South Africa will receive one due to social contraindications. To qualify for a transplant, social and socioeconomic criteria are used as exclusion criteria for patients as transplant requires adherence to lifelong treatment and the presence of social support structures for positive outcomes. Our sensitivity analysis shows that the cost-effectiveness of vaccination was highly sensitive to varying access to liver transplant. When increasing the access to liver transplant to 100% for the baseline and Scenario 1, the ICER for Scenario 1 becomes cost-effective against the CET (ICER = USD 2425). Given these findings, we recommend further research is conducted to understand the access to liver transplants in South Africa to better estimate the cost of liver transplant care for hepatitis A patients and cost-effectiveness of vaccination.

The main strength of this study is that, to the best of our knowledge, it is the first to utilize a dynamic modelling approach to understand the epidemiology of hepatitis A in South Africa and to conduct a cost-effectiveness analysis of routine hepatitis A vaccination in the country. Our study uses local cost data drawn from a retrospective folder review of hepatitis A cases requiring outpatient care or hospitalization in South Africa and this contextually relevant data leads to the derivation of more realistic cost projections in the country.

The modelling presented in this paper has been used to develop a user-friendly application for vaccine policy makers to further interrogate the model outcomes and consider the costs and benefits of introducing routine hepatitis A vaccination in South Africa. The application allows users to vary clinical parameters in the model such as the proportion of hepatitis A patients that require hospitalization or develop viral-induced liver failure as well as associated costs. Once the user has varied these parameters, they have the opportunity to develop vaccination programs and compare outcomes to assess the potential cost-effectiveness. The application has been developed in R using the Rshiny package and can be accessed using this link (https://masha-app.shinyapps.io/HepA-VacExplorer/ (accessed on 21 January 2024)).

Several limitations must be considered in the interpretation of our results from the hepatitis A transmission model. It is important to take into account that incidence rates for hepatitis A are likely underreported due to the circumstances and mild nature with which the disease can present. In addition, the transmission model assumes that all symptomatic cases seek treatment for infection, which may not be the case. As these estimates were missing from the literature, we recommend more research be conducted on treatment seeking behaviors for patients with hepatitis A.

It should also be noted that the projected increase in hepatitis A seroprevalence among children < 10 years old in South Africa is unexpected and these results should be interpreted with caution. While the model was calibrated using the largest description of HAV seroprevalence within South Africa to date, the HAV seroprevalence data published by the NICD were unable to determine yearly seroprevalence trends due to the low volumes of anti-HAV total antibody testing and uneven distribution among age groups [14]. The data that we used to calibrate the model was available only until 2015, which means caution should be applied when interpreting forecasted results until 2030. In addition, we were unable to determine a trend in the environmental presence of HAV which plays a large part in childhood hepatitis A transmission. To validate and update the model’s seroprevalence projections, new data on anti-HAV IgG and IgM positivity and the environmental presence of HAV in South Africa should be included in the model as it comes available. Further analysis should include fitting the model to a decreasing trend in HAV seroprevalence between 2005 and 2015. Other limitations of this study include that the cost of hepatitis A inpatient treatment is likely overestimated as it is drawn from a tertiary hospital setting.

## 5. Conclusions

The results of this study indicate that implementation of a single dose of the hepatitis A vaccine in South African children < 2 years old between 2023 and 2030 generates health gains in comparison to the baseline approach, however, is not cost-effective against the CET with an ICER per DALY averted of USD 21,006. Given the sensitivity of the model to varying access to liver transplant, we recommend further research is conducted to understand the access parameters in order to better inform considerations of hepatitis A vaccination policies. In addition, further analysis using this model might include fitting the model to a decreasing trend in HAV seroprevalence between 2005 and 2015.

## Figures and Tables

**Table 1:** Model compartments and description.

Abbreviation	Compartment	Description
M	Maternal antibodies	Presence of maternally acquired anti-HAV IgG antibodies
S	Susceptible	No presence of anti-HAV IgG antibodies
E	Exposed	Exposed to the hepatitis A virus with the risk of infection
A	Asymptomatic	Infected with the hepatitis A virus following the incubation period
Sy	Symptomatic	Infected with the hepatitis A virus following the incubation period
O	Outpatient case	Hepatitis A case requiring outpatient care at a tertiary level facility
H_i_	Hospitalized infectious case	Hepatitis A case requiring hospitalization at a tertiary level facility while shedding HAV
H_n_	Hospitalized non-infectious case	Hepatitis A case requiring hospitalization at a tertiary level facility after shedding HAV
N	Recovering case	Hepatitis A case with waning anti-HAV IgM antibodies
R	Recovered and immune	Previous hepatitis A case with anti-HAV IgG antibodies developed through infection
ALF	Viral-induced acute liver failure	Hepatitis A case resulting in acute liver failure defined as the development of encephalopathy and synthetic function impairment following acute liver injury in an individual without pre-existing liver disease
ALF_R_	Spontaneous recovery from acute liver failure	Viral-induced acute liver case that recovers without liver transplant
ALF_D_	Death due to acute liver failure	Viral-induced acute liver case that dies due to any cause
ALF_T_	Liver transplant case	Viral-induced acute liver transplant case that requires liver transplant for recovery
T_R_	Liver transplant recovery	Viral-induced acute liver transplant case that requires and receives liver transplant
T_D_	Liver transplant death	Liver transplant case that dies due to any cause
D	Hepatitis A death	Hepatitis A case that dies due to any cause
V	Vaccinated	Vaccinated with one or two doses of hepatitis A vaccine with sufficient development of anti-HAV IgG antibodies for protection against infection

**Table 2:** Parameter values and distributions.

Parameter	Symbol	Baseline Value or Fitted Range When Stated [Uncertainty Distribution/Range]	Source
Proportion of population born with maternal anti-HAV antibodies	*propM*	t ≤ 2005: 0.72 t = 2006: 0.76 t = 2007: 0.79 t = 2008: 0.81 t = 2009: 0.77 t = 2010: 0.77 t = 2011: 0.76 t = 2012: 0.71 t = 2013: 0.66 t = 2014: 0.63 t ≥ 2015: 0.64	Calculated based on annual female population aged 15–49, fertility rates for ages 15–49, age specific annual HAV seroprevalence rates for ages 15–49, and annual birth rates
Rate of maternal anti-HAV antibody waning (years)	*tau*	1	Guzelkucuk et al., 2019 [15]
Incubation period (days)	*nu*	28 [15, 50]	Foster et al., 2021 [16]
Probability of asymptomatic hepatitis A infection in age group_i_	*propA_i_*	i ≤ 6: 0.7 i ≥ 7: 0.3	Foster et al., 2021 [16]
Probability of outpatient care due to hepatitis A infection in age group_i_	*propO_i_*	i ≤ 12 = 0.68 13 ≥ i ≤ 14: 0.7262 15 ≥ i ≤ 16: 0.6662 17 ≥ i ≤ 19: 0.7362	Calculated as (1−propH+propF)
Probability of hospitalization due to hepatitis A infection in age group_i_	propH_i_	i ≤ 12 = 0.21 13 ≥ i ≤ 14: 0.17 15 ≥ i ≤ 16: 0.23 17 ≥ i ≤ 19: 0.16	Canuel et al., 2007 [17]
Probability of viral-induced acute liver failure in age group_i_	*propF_i_*	i ≤ 12 = 0.11 i > 12 = 0.1038	Keles et al., 2021 & Jiang et al., 2018 [18,19]
Probability of spontaneous recovery from acute liver failure in age group_i_	*propFr_i_*	0.25	Mendizabal et al., 2016 [20]
Probability of liver transplant due to hepatitis A infection in age group_i_	propT_i_	0.26	Mendizabal et al., 2016 [20]
Probability of death due to acute liver failure in age group_i_	*propFD_i_*	0.49	Mendizabal et al., 2016 [20]
Probability of death due to liver transplant in age group_i_	*propTD*	0.16	Mendizabal et al., 2016 [20]
Recovery from hepatitis A infectious period (days)	*gamma*	21 [14, 180]	Foster et al., 2021 [16]
Days for hepatitis A cases to seek care	*trt*	2 [1, 3]	Patterson et al., 2022 [21]
Days for hospitalized hepatitis A cases to develop acute liver failure	*Frate*	2 [1, 3]	Patterson et al., 2022 [21]
Days for acute liver failure cases to die	*FDrate*	16 [1, 20]	Allen et al., 2016 [22]
Days for acute liver failure cases to spontaneously recover	*gammaF*	21 [14, 180]	John Hopkins 2021 [23]
Days for acute liver failure cases to be diagnosed as liver transplant cases	*Trate*	3 [1, 10]	Allen et al., 2016 [22]
Days for liver transplant cases to die (years)	*TDrate*	1	Based on mortality probabilities reported annually
Days for liver transplant cases to recover	gammaT	21 [14, 180]	John Hopkins 2021 [23]
Days for hepatitis A cases to lose IgM antibodies and develop IgG antibodies marking immunity (months)	*Rrate*	180 [90, 365.25]	Prabdial-Sing et al., 2021 [14]
Person-to-person contact scaling factor	*Prel*	0.002 [0, 0.01]	Calibrated to fit national HAV seroprevalence data set
Person-to-environment contact scaling factor	*Erel*	0.0007 [0, 0.01]	Calibrated to fit national HAV seroprevalence data set
Prevalence of hepatitis A in environment	*PrevE*	t = 2005: 0.3 [0, 1] 2005 > t ≤ 2010: 0.5 [0, 1] t > 2010: 0.8 [0, 1]	Calculated from supplementary data files associated with Kuodi et al., 2020 [24]
Age-specific number of infective contacts per year	*betaE_i_*	i:1= 1084.79 i:2 = 1139.04 i:3 = 813.61 i:4 = 678.02 i:5 = 542.42 i:6 = 813.66 i:7 = 542.42 i:8 = 271.29 i:9 = 105.90 i:10 = 2169.59 i:11 = 189.84 i:12 = 162.72 i:13 = 678.02 i:14 = 542.42 i:15 = 406.83 i:16 = 271.24 i:17 = 135.64 i:18 = 52.96 i:19 = 52.96	Baseline values from Venter et al., 2007 calibrated to fit national HAV seroprevalence data set [25]

**Table 5:** Impact of modelled vaccination scenarios on the burden of hepatitis A (2023–2030).

Scenario	Number of Vaccines Required	Symptomatic Cases Averted	Deaths Averted
1	5.3 million	136,042 [IQR: 88,842–221,483]	31,106 [IQR: 22,975–36,742]
2	7.8 million	255,857 [IQR: 159,721–225,065]	31,585 [IQR: 23,388–37,240]
3	9.2 million	259,318 [IQR: 162,828–477,574]	30,982 [IQR: 22,502–37,488]
4	11.7 million	267,947 [IQR: 169,625–482,796]	29,890 [IQR: 21,235–37,309]

**Table 6:** Cost-effectiveness of modelled scenarios referencing across a common baseline (2023–2030).

Scenario	Total Costs	Incremental Costs	Total DALYs	DALYs Averted	Incr. Cost per DALY Averted
Baseline	USD 1,530,392,760 [IQR: USD 1,062,167,392 to USD 2,348,837,236]	---	27,137	---	---
1	USD 1,714,015,277 [IQR: USD 1,382,389,882 to USD 2,435,383,515]	USD 183,622,517	18,396	8741	USD 21,007
2	USD 2,009,207,209 [IQR: USD 1,676,218,304 to USD 2,733,706,843]	USD 478,814,449	18,266	8871	USD 53,975
3	USD 2,195,073,864 [IQR: USD 1,862,640,398 to USD 2,904,961,085]	USD 664,681,104	18,440	8697	USD 76,426
4	USD 2,851,373,642 [IQR:USD 2,447,209,061 to USD 3,478,490,923]	USD 1,320,980,882	19,151	7986	USD 165,412

The incremental costs and DALYs averted presented in this table are calculated by referencing across the common baseline. Abbreviations: Incr. = incremental; DALYs = Disability adjusted life years.

## Data Availability

The raw data supporting the conclusions of this article can be requested from the National Health Laboratory Service of South Africa.

## Supplementary Materials

The following supporting information can be downloaded at: https://www.mdpi.com/article/10.3390/vaccines12020116/s1, Table S1: Ordinary differential equations; Table S2: Daily contact matrix; Table S3: Cost-effectiveness of modelled scenarios referencing previous undominated approach (2023–2030). Table S4: One-way sensitivity analysis for Scenario 1 ICER Results.
