# Supplementary material for: Modelling the Cost-Effectiveness of Hepatitis A in South Africa
Source: Vaccines (Basel). 2024 Jan 24;12(2):116. doi: 10.3390/vaccines12020116 (PMC10893480; doi:10.3390/vaccines12020116)
Supplement: Supplementary file 1 [file vaccines-12-00116-s001.zip › 2023 10 22_Hepatitis A Manuscript_Vaccine_Supplementary File.pdf]

Supplementary Table S1: Ordinary differential equations

$$\frac{dM}{dt} = propM * mu * N - tau * M - d_1 * M - (1 - d_1) * age_1 * M$$

$$\frac{dS_i}{dt} = (1 - propM) * mu * N + tau * M - propV * S_i * Vrate * (1 - propVF) - lambda_i * S_i - d_i * S_i - (1 - d_i) * age_i * S_i$$

$$\frac{dV_i}{dt} = propV * S_i * Vrate * (1 - propVF) - d_i * V_i - (1 - d_i) * age_i * V_i$$

$$\frac{dE_i}{dt} = lambda_i * S_i - nu * propA_i * E_i - nu * (1 - propA_i) * E_i - d_i * E_i - (1 - d_i) * age_i * E_i$$

$$\frac{dA_i}{dt} = nu * propA_i * E_i - gamma * A_i - d_1 * A_i - (1 - d_i) * age_i * A_i$$

$$\frac{dSy_i}{dt} = nu * (1 - propA_i) * E_i - trt * propO_i * Sy_1 * trt * propH_i * Sy_1 - propF_i * trt * Sy_1 - d_i * Sy_i - (1 - d_i) * age_i * A_i$$

$$\frac{dO_i}{dt} = trt * propO_i * Sy_i - gamma * O_i - d_i * O_i - (1 - d_i) * age_i * O_i$$

$$\frac{dHi_i}{dt} = trt * propH_i * Sy_i - gamma * Hi_i - d_i * Hi_i - (1 - d_i) * age_i * Hi_i$$

$$\frac{dHn_i}{dt} = gamma * Hi_i - gamma * Hn_i - d_i * Hn_i - (1 - d_i) * age_i * Hn_i$$

$$\frac{dALF_i}{dt} = propF_i * trt * Sy_i - (1 - propT_i) * propFD_i * Frate * Fu_i - propFR_i * Frate * Fu_i - propT_i * Frate * Fu_i - d_i * Fu_i - (1 - d_i) * age_i * Fu_i$$

$$\frac{dALFd_i}{dt} = propFD_i * Frate * ALF_i - FDrate * ALFd_i - d_i * ALFd_i - (1 - d_i) * age_i * ALFd_i$$

$$\frac{dALFr_i}{dt} = propFr_i * Frate * Fu_i - gammaF * ALFr_i - d_i * ALFr_i - (1 - d_i) * age_i * ALFr_i$$

$$\frac{dALFt_i}{dt} = propT_i * Frate * Fu_i - propTD_1 * Trate * ALFt_i - (1 - propTD_i) * rate * T_i - d_i * T_i - (1 - d_i) * age_i * ALFt_i$$

$$\frac{dTd_i}{dt} = propTD_i * Trate * T_i - TDrate * Td_i - d_i * Td_i - (1 - d_i) * age_i * Td_i$$

$$\frac{dTr_i}{dt} = (1 - propTD_i) * Trate * T_i - gammaT * Tr_i - d_i * Tr_i - (1 - d_i) * age_i * Tr_i$$

$$\frac{dD_i}{dt} = TDrate * Td_i + FDrate * ALFd_i$$

$$\frac{dN_i}{dt} = gamma * A_i + gamma * O_i + gamma * Hn_i - Rrate * N_i - d_i * N_i - (1 - d_i) * age_i * N_i$$

$$\frac{dR_i}{dt} = Rrate * N_i + gammaF * Fr_i + gammaT * Tr_i - d_i * R_i - (1 - d_i) * age_i * R_i$$

Supplementary Table S2: Daily contact matrix

		Age																		
Age		0	1	2	3	4	5	6	7	8	9	10 to 14	15t o 19	20 to 29	30 to 39	40 to 49	50 to 59	60 to 69	70 to 79	80+
	0	0.13	0.13	0.13	0.12	0.12	0.13	0.13	0.13	0.12	0.12	0.17	0.11	0.46	0.52	0.20	0.13	0.06	0.02	0.00
	1	0.13	0.13	0.12	0.12	0.12	0.13	0.13	0.13	0.12	0.12	0.17	0.11	0.46	0.52	0.19	0.13	0.06	0.02	0.00
	2	0.13	0.12	0.12	0.12	0.12	0.13	0.13	0.13	0.12	0.12	0.17	0.11	0.45	0.51	0.19	0.13	0.06	0.02	0.00
	3	0.12	0.12	0.12	0.12	0.12	0.12	0.12	0.12	0.12	0.12	0.17	0.11	0.45	0.51	0.19	0.13	0.06	0.02	0.00
	4	0.12	0.12	0.12	0.12	0.12	0.12	0.12	0.12	0.12	0.11	0.17	0.11	0.44	0.50	0.19	0.13	0.06	0.02	0.00
	5	0.06	0.06	0.06	0.06	0.06	0.06	0.06	0.06	0.06	0.05	0.41	0.12	0.27	0.47	0.26	0.09	0.05	0.01	0.00
	6	0.06	0.06	0.06	0.06	0.06	0.06	0.06	0.06	0.06	0.06	0.41	0.12	0.28	0.48	0.26	0.09	0.05	0.01	0.00
	7	0.06	0.06	0.06	0.06	0.06	0.06	0.06	0.06	0.06	0.05	0.41	0.12	0.27	0.47	0.26	0.09	0.05	0.01	0.00
	8	0.06	0.06	0.06	0.05	0.05	0.06	0.06	0.06	0.05	0.05	0.40	0.11	0.27	0.46	0.25	0.09	0.05	0.01	0.00
	9	0.05	0.05	0.05	0.05	0.05	0.05	0.06	0.05	0.05	0.05	0.38	0.11	0.26	0.44	0.24	0.09	0.05	0.01	0.00
	10 to 14	0.12	0.12	0.12	0.11	0.11	0.12	0.12	0.12	0.11	0.11	12.55	1.31	1.22	1.60	1.46	0.42	0.17	0.07	0.02
	15 to 19	0.07	0.07	0.07	0.07	0.07	0.07	0.07	0.07	0.07	0.07	3.74	9.68	3.30	1.56	1.75	0.58	0.16	0.04	0.01
	20 to 29	0.13	0.13	0.13	0.13	0.12	0.13	0.13	0.13	0.12	0.12	0.95	5.04	16.20	6.20	3.84	2.04	0.53	0.08	0.02
	30 to 39	0.24	0.24	0.23	0.23	0.23	0.24	0.24	0.24	0.23	0.22	2.37	1.38	6.41	8.32	4.64	1.84	0.68	0.10	0.02
	40 to 49	0.23	0.22	0.22	0.22	0.21	0.22	0.23	0.22	0.22	0.21	2.25	2.50	3.75	4.94	5.00	1.79	0.53	0.10	0.02
	50 to 59	0.20	0.20	0.20	0.19	0.19	0.20	0.20	0.20	0.19	0.19	1.93	1.89	3.57	3.18	3.11	2.19	0.67	0.11	0.03
	60 to 69	0.14	0.14	0.14	0.14	0.13	0.14	0.14	0.14	0.14	0.13	1.11	0.84	1.92	2.61	1.84	1.33	0.96	0.18	0.02
	70 to 79	0.08	0.08	0.08	0.08	0.08	0.08	0.09	0.08	0.08	0.08	1.20	0.95	0.65	0.98	1.13	0.77	0.56	0.34	0.09
	80+	0.10	0.09	0.09	0.09	0.09	0.10	0.10	0.09	0.09	0.09	0.60	0.48	0.26	0.41	0.51	0.38	0.19	0.12	0.04

Supplementary Table S3: Cost-effectiveness of modelled scenarios referencing previous undominated approach (2023-2030)

Scenario	Total Costs	Incremental Costs	Total DALYs	DALYs averted	Incr. Cost per DALY averted
Baseline	$1,530,392,760		27,137		
1	$1,714,015,277	$183,622,517	18,396	8,741	$21,007
2	$2,009,207,209	$295,191,932	18,266	130	$2,270,707
3	$2,195,073,864	$185,866,655	18,440	-174	($1,068,199)
4	$2,851,373,642	$656,299,778	19,151	-711	($923,066)

The Incremental costs and DALYs averted presented in this table are calculated by referencing the previous undominated and less costly scenario.

Abbreviations: Incr. = incremental; DALYs = Disability adjusted life years

Supplementary Table S4: One-way sensitivity analysis for Scenario 1 ICER Results

One-way sensitivity analysis	Scenario 1 Total Cost	DALYS averted against baseline	Incr. cost per DALY averted against baseline
Cost of clinic visit removed	$1,128,653,105	18,396	$45,958
Access to liver transplant at 0%	$1,531,224,497	18,396	-$31,048
Access to liver transplant at 100%	$2,140,527,097	18,396	$2,426
Discount rate at 0%	$2,025,301,242	20,984	-$19,972
Discount rate at 10%	$1,477,986,262	16,406	-$22,079
